# Silver Nanoparticles and Mitochondrial Interaction

**DOI:** 10.1155/2013/312747

**Published:** 2013-09-11

**Authors:** Eriberto Bressan, Letizia Ferroni, Chiara Gardin, Chiara Rigo, Michele Stocchero, Vincenzo Vindigni, Warren Cairns, Barbara Zavan

**Affiliations:** ^1^Department of Biomedical Sciences, University of Padova, Via G. Colombo 3, 35100 Padova, Italy; ^2^Department of Neurosciences, University of Padova, Via Venezia 90, 35100 Padova, Italy; ^3^CNR-IDPA c/o Department of Environmental Sciences Informatics and Statistics, Universita Ca' Foscari, Dorsoduro 2137, 30123 Venezia, Italy

## Abstract

Nanotechnology has gone through a period of rapid growth, thus leading to the constant increase in the application of engineered nanomaterials in daily life. Several different types of nanoparticles have been engineered to be employed in a wide array of applications due to their high surface to volume ratio that leads to unique physical and chemical properties. So far, silver nanoparticles (AgNps) have been used in many more different medical devices than any other nanomaterial, mainly due to their antimicrobial properties. Despite the promising advantages posed by using AgNps in medical applications, the possible health effects associated with the inevitable human exposure to AgNps have raised concerns as to their use since a clear understanding of their specific interaction with biological systems has not been attained yet. In light of such consideration, aim of the present work is the morphological analysis of the intracellular behavior of AgNps with a diameter of 10 nm, with a special attention to their interaction with mitochondria.

## 1. Introduction

Antibacterial properties of silver ions are well known. Indeed, silver has been used since time immemorial in different chemical forms to treat burns, wounds, and several different infections caused by pathogenic bacteria. Interestingly, for thousands of years, silver and silver ions have been used for their bactericidal properties [1, 2], which include

1. multilevel antibacterial effects that considerably reduce the chances of developing resistance since this effect of silver is thought to be due to blockage of respiratory enzyme pathways and alteration of microbial DNA and the cell wall [3, 4];
2. effectiveness against multidrug-resistant organisms [5, 6];
3. low systemic toxicity [7, 8].

Over the past decade, a variety of advanced silver-based medical devices have been developed with considerable variations in the structure, composition, and silver content. In recent years, nanotechnology has provided the means of producing pure silver nanoparticles, markedly increasing the rate of silver ion release and its antimicrobial activity as well.

The use of oral implants in the rehabilitation of partially and fully edentulous patients is widely accepted even though failures do occur [9]. The chance for implants to integrate can for example be jeopardised by the intraoral presence of bacteria and concomitant inflammatory reactions. The longevity of osseointegrated implants can be compromised by occlusal overload and/or plaque-induced peri-implantitis, depending on the implant geometry and surface characteristics. Animal studies, cross-sectional and longitudinal observations in man, and association studies indicate that peri-implantitis is characterised by a microbiota comparable to that of periodontitis (high proportion of anaerobic Gram-negative rods, motile organisms, and spirochetes), but this does not necessarily prove a causal relationship [10]. However, in order to prevent such a bacterial shift, the following measures can be considered: periodontal health in the remaining dentition (to prevent bacterial translocation), the avoidance of deepened peri-implant pockets, and the use of a relatively smooth abutment and implant surface. Finally, periodontitis enhancing factors such as smoking and poor oral hygiene also increase the risk for peri-implantitis [11].

The oral cavity is populated by a variety of microorganisms. The microbial communities in the oral cavity are polymicrobial and exist primarily as biofilms [12]. These biofilms can be responsible for several local diseases, including periodontal and peri-implant diseases, which can lead to the loss of teeth or implants, respectively. The potential of silver nanoparticles (AgNps) to reduce bacterial adhesion to dental implant surfaces and to prevent biofilm formation has been investigated by many authors [13, 14] with a view to reducing the risk of peri-implant infections. Another interesting application of Ag NPs in dentistry pertains the structural and surface modification of bone grafts and membranes with a view to preventing the risk of contamination and associated infection that are common when bone augmentation techniques such as guided bone regeneration (GBR) and guided tissue regeneration (GTR) are used [15–19].

Despite the widespread use of Ag NPs, a lack of information on their biological effects on human cells and environments still exists. Some authors have investigated the potential toxicity of Ag NPs in different cell systems, including bacteria and mammalian cells. Such studies have attributed the cytotoxicity of Ag NPs to several possible mechanisms, including the dissolving or release of Ag ions from the nanoparticles, the disruption of cell membrane integrity, oxidative stress, protein or DNA binding and damage, the generation of reactive oxygen species, and apoptotic cell death. The toxic mechanism seems likely to depend on the nanoparticles' properties too, for example surface area, size and shape, capping agent, surface charge, particle purity, structural distortion, and the bioavailability of the individual particles. To this view, in previous works, our group compared the silver structure and content of several Ag dressing based products, focusing main attention on Acticoat [20, 21].

Acticoat is a nanocrystalline silver dressing composed of two layers of silver-coated high-density polyethylene, enclosing a rayon/polyester core of apertured nonwoven fabric. The elements that compose Acticoat are welded together ultrasonically. When moistened with water, microscopic nanocrystals of metallic silver are released from the dressing onto the wound bed. The silver has an antimicrobial action which destroys a range of bacteria, including both Gram-positive and Gram-negative bacteria [22].

SEM images of this product showed that the polyethylene fibers are coated with Ag. The coating appears homogeneous and uniform. The size of the Ag nanocrystals was determined at a magnification of 50,000; the images show that the particle size ranges from 200 to 450 nm. 

Moreover, its cytotoxicity was tested *in vitro* and *in vivo* on human. In the present work the studies are carried on focusing on the intracellular behavior of Ag nanoparticles released form acticoat during a wound dressing.

## 2. Material and Methods

### 2.1. Human Skin Samples

Patients were eligible for the study if recruited <24 h postburn injury and were affected by partial-thickness burns. Patients were excluded if they were affected by full thickness burns or had a compromised immune system or were known to be hypersensitive to silver and its compounds.

Patients were also excluded in case of comorbidity (e.g., diabetes and cardiac or renal disease), chemical or electrical burns, multiple trauma, or were aged <5 or >60. Skin biopsies were obtained from a set of eligible patients who gave consent for taking biopsy materials for scientific purposes, and the study was performed in compliance with the Declaration of Helsinki ethical guidelines.

Biopsies were collected by using punches of 4 mm inner diameter 7 mm depth. After seven days of treatment at dressing removal, two more duplicates were taken, one from the healed area and another from an unhealed zone. After 10 more days of treatment with a new dressing, another duplicate sample was taken from the newly healed area.

### 2.2. TEM

The samples were preserved in a 2.5% glutaraldehyde/0.1 M sodium cacodylate buffer overnight at 4°C. The samples were then treated with 1% OsO4/0.1 M sodium cacodylate buffer and dehydrated using ethanol solutions of increasing concentrations before embedding in EPON epoxy resins. Ultrathin sections (ultramicrotome, LKB, Stockholm, Sweden) were obtained and treated with 1% uranyl acetate and 1% lead citrate. The samples were analyzed by TEM (Electronic Microscopy Service, Department of Biology, University of Padova, Padua, Italy) using a Tecnai G12 electron microscope (FEI, acceleration voltage 100 kV). The image acquisition system consisted of a video camera, TIETZ (Tietz Video and Image Processing Systems GmbH, Gauting, Germany), and the TIA FEI Imaging Software (FEI Company, Hillsboro, OR, USA).

### 2.3. Cell Cultures

Human dermal fibroblasts were prepared according to a modified version of the Rheinwald and Green protocol. After epithelial sheet dispase removal, the dermis was cut into small pieces (2-3 mm^2^), and fibroblasts were isolated by sequential digestion with 0.25% w/v trypsin for 20 min and 0.25% w/v collagenase for 4 h. These cells were then cultured with Dulbecco's Modified Eagle Medium (DMEM), (Lonza S.r.l., Milano, Italy) supplemented with 10% fetal bovine serum (FBS) (Bidachem S.p.A., Milano, Italy) and 100 units/mL penicillin and 100 *μ*g/mL streptomycin to form complete DMEM (cDMEM). The medium was changed twice a week, and the cells were harvested by trypsin treatment. After detachment from culture plates, fibroblasts were cultured in 3D collagen-based scaffolds (MatriDerm, Dr. Suwelack Skin and Health Care AG, Billerbeck, Germany) at a density of 10^5^ cells/cm^2^, obtaining a reconstructed dermal-like tissue *in vitro*. Cells were grown in the 3D scaffold for 10 days in 800 *μ*L of cDMEM. The Ag NP-based dressing was applied above the 3D cell cultures.

### 2.4. MTT Assay

To determine the kinetics of cell growth with or without Ag NPs, the MTT-based (methyl-thiazolyl-tetrazolium) cytotoxicity assay was performed according to the method of Denizot and Lang with minor modifications. This colorimetric assay is an indirect method for assessing cell growth and proliferation. MTT gives a yellowish aqueous solution, which, on reduction with dehydrogenases or reducing agents present in metabolically active cells, yields a violet-blue water insoluble dye compound, formazan. The lipid soluble formazan is extracted with organic solvents and quantified spectrophotometrically. The amount of MTT formazan produced is directly proportional to the metabolic activity of cells. After harvesting the culture medium, the cells were incubated for 3 h at 37°C in 1 mL of 0.5 mg/mL MTT solution prepared in phosphate buffer saline solution (PBS). After removal of the MTT solution by pipette, 0.5 mL of 10% dimethyl sulfoxide in isopropanol (iDMSO) was added to extract the formazan in the samples for 30 min at 37°C. For each sample, absorbance values at 570 nm were recorded in duplicate on 200 *μ*L aliquots deposited in microwell plates using a multilabel plate reader (Victor 3 Perkin Elmer, Milano, Italy). The mitochondrial functionality in the Ag NP-treated cells is calculated as the ratio between the absorbance at 570 nm of the treated sample and the absorbance of a control sample expressed as a percentage.

### 2.5. Morphological Analysis

Concurrently with the MTT assay, morphological analyses were carried out on the duplicate samples. After removing the Ag NP-based dressing and the culture medium, the remaining dermal-like tissue was embedded in Optimal Cutting Temperature (OCT) compound, frozen in liquid nitrogen and preserved at −80°C until cutting. Tissue sections (>7 *μ*m thickness) were obtained using a cryostat (CM1950, Leica, Milano, Italy) and deposited onto gelatin-coated glass slides. They were fixed with absolute acetone for 10 min at room temperature and cryopreserved at −20°C until use. In order to visualize the cell distribution inside the scaffold and to investigate the possibility of nuclear fragmentation, the fibroblasts nuclei were stained with H33342 fluorochrome (Sigma Aldrich, Milano, Italy, final concentration of 2 *μ*g/mL). The samples were observed using a Zeiss Axioplan fluorescence microscope equipped with a digital camera (DC500, Leica, Milano, Italy).

In order to quantify the number of live cells and highlight the presence of apoptotic cells, a parallel set of *in vitro* experiments were carried out. Hoechst H33342 dye was added to the 3D dermal-like cell culture simultaneously with YO-PRO-1 iodide dye (excitation wavelength 491 nm/emission wavelength 509 nm, Molecular Probes). Hoechst H33342 dye stains the nuclei in the whole population of cells, while YO-PRO-1 stains specifically the apoptotic cells. YO-PRO-1 is a green fluorescent probe, which can enter cells once their plasma membrane has reached a certain degree of permeability.

The cell membrane during apoptosis becomes slightly permeable and YO-PRO-1 can freely enter the cell and bind to its nucleic acids, enhancing its fluorescence intensity. The number of different cells was counted, and live cells are calculated as the difference between the number of cells stained with Hoechst H33342 and the number of apoptotic cells stained with YO-PRO-1. Immediately after the removal of the Ag NP-based dressing from the 3D cell cultures, Hoechst 33342 and YO-PRO-1 were added to the cell cultures. The cells were incubated at 37°C for one hour, and then, the culture multiwell plate containing the cells was transferred to a confocal laser scanning microscope to monitor YO-PRO-1 and Hoechst fluorescence.

A fluorescence confocal laser scanning microscope (Axiovert 100M, Zeiss, Germany) with a 10° magnification objective was used for the detection of Hoechst H33342 and YO-PRO-1 stained cells. The fluorescent dye, YO-PRO-1, was excited with a 25 mW Argon laser at 488 nm. Emission was recorded above 510 nm. The Hoechst H33342 fluorescence was detected at 460 nm after excitation at 346 nm. The microscope was equipped with a motorized stage, and the LSM 510 (Zeiss) software enabled memorization of stage positions. For each sample, images were taken at the preset stage positions at various depths.

### 2.6. ROS Measurements

**Figure 1:**
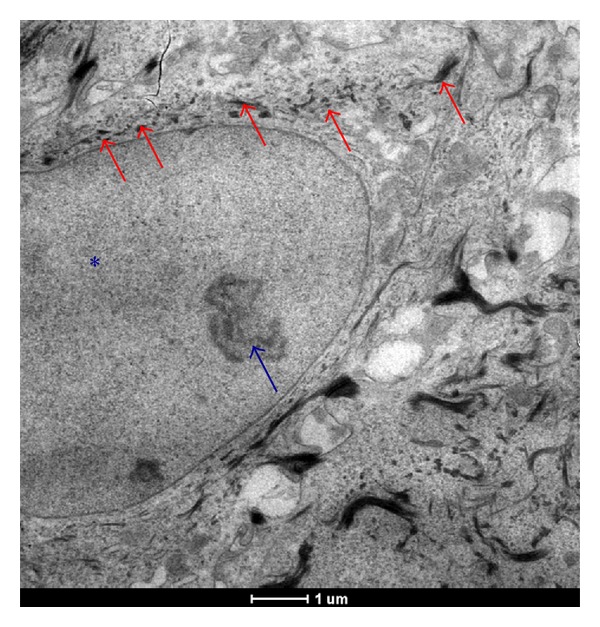
TEM images of a fibroblast present in a healed skin sample treated with AgNP based medical devices. The cells are healthy as the right morphological features show: integrity of plasma membrane, nuclear integrity (blue^*∗*^), and active nucleolus (blue arrow).

The OxiSelect ROS Assay Kit is a cell-based assay for measuring hydroxyl, peroxyl, and other reactive oxygen species activity within a cell. The assay employs the cell-permeable fluorogenic probe DCFH-DA, which diffuses into cells and is deacetylated by cellular esterases into the nonfluorescent DCFH (Figure 1). In the presence of ROS, DCFH is rapidly oxidized to highly fluorescent DCF. Fluorescence is read on a standard fluorometric plate reader.

## 3. Results and Discussion

fibroblast present in the deeper layer of burned skin are able to uptake AgNps (Figure 1, red arrows). The cells are healthy as the right morphological features show: integrity of plasma membrane, nuclear integrity (blue*), and active nucleolus (blue arrow). All nanoparticles are taken up by mammalian cells by such mechanisms as pinocytosis, endocytosis dependent on caveolae and lipid raft composition, clathrin-dependent endocytosis and phagocytosis [10], endocytosis {Endo (within) cytosis (cell)}, is a process in which a substance gains entry into a cell without passing through the cell membrane. This process is subdivided into three different types: pinocytosis, phagocytosis, receptor mediatedendocytosis. Receptor mediated endocytosis is an endocytotic mechanism in which specific molecules are ingested into the cell. The specificity results from a receptor-ligand interaction. Receptors on the plasma membrane of the target tissue will specifically bind to ligands on the outside of the cell. An endocytotic process occurs, and the ligand is ingested. In each case, endocytosis results in the formation of an intracellular vesicle by virtue of the invagination of the plasma membrane and membrane fusion.

**Figure 2:**
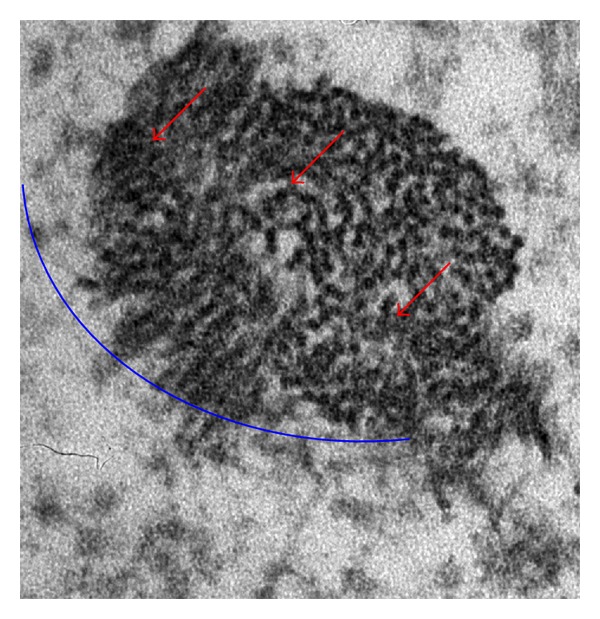
TEM images of a fibroblast present in a healed skin sample treated with AgNP based medical devices. Nanoparticles taking up by endocytosis. AgNps in normal human dermal fibroblasts take up AgNps (red arrows) by endocytosis (blue arc).

**Figure 3:**
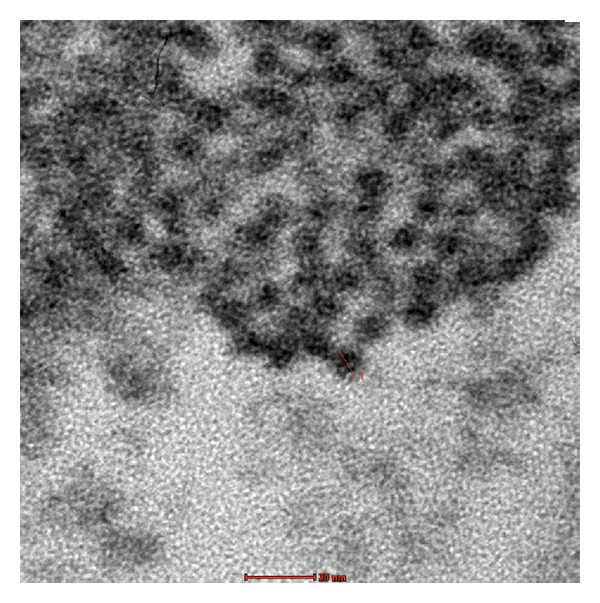
TEM images of AgNps (red arrows) in cytoplasmatic space of fibroblast present in a healed skin sample treated with AgNP based medical devices. The diameter of particles released from the medical devices are of 20 nm, indicating that their aggregates are able to enter the cells by endocytosis.

AgNps are no exception in this respect; as shown in Figure 2, indeed, normal human dermal fibroblasts take up AgNps (red arrows) by endocytosis (blue arc). As well reported in Figure 3 the diameter of particles released from the medical devices is of 20 nm, (note: a fibroblast size is about 100 *μ*m) indicating that their aggregates are able to enter into the cells by endocytosis.

**Figure 4:**
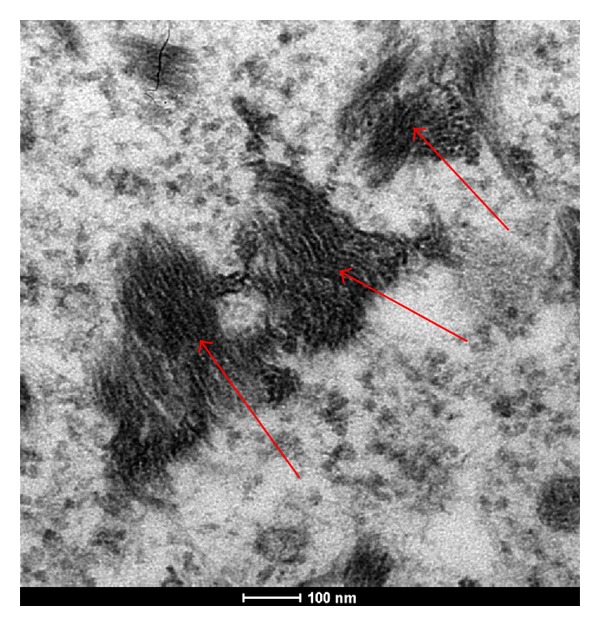
TEM images of AgNps (red arrows) in cytoplasmatic space of fibroblast present in a healed skin sample treated with AgNP based medical devices.

**Figure 5:**
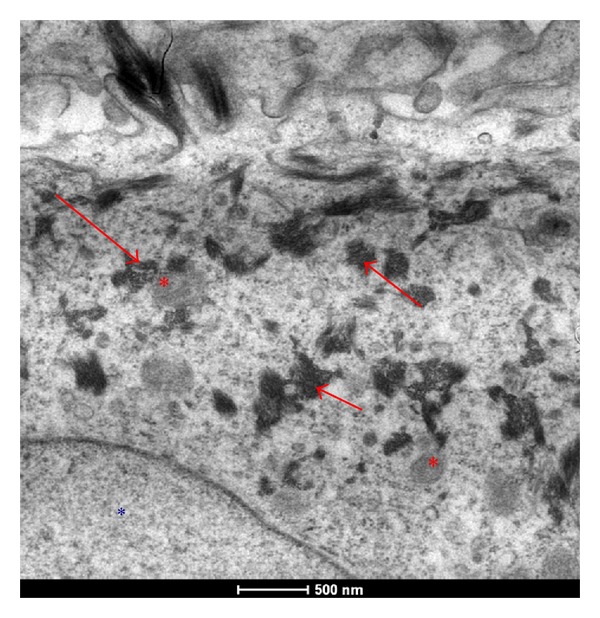
TEM images of AgNps (red arrows) in cytoplasmatic space of fibroblast present in a healed skin sample treated with AgNP based medical devices. AgNP are closed to the mitochondria (red*); nuclei is indicated by blue*.

**Figure 6:**
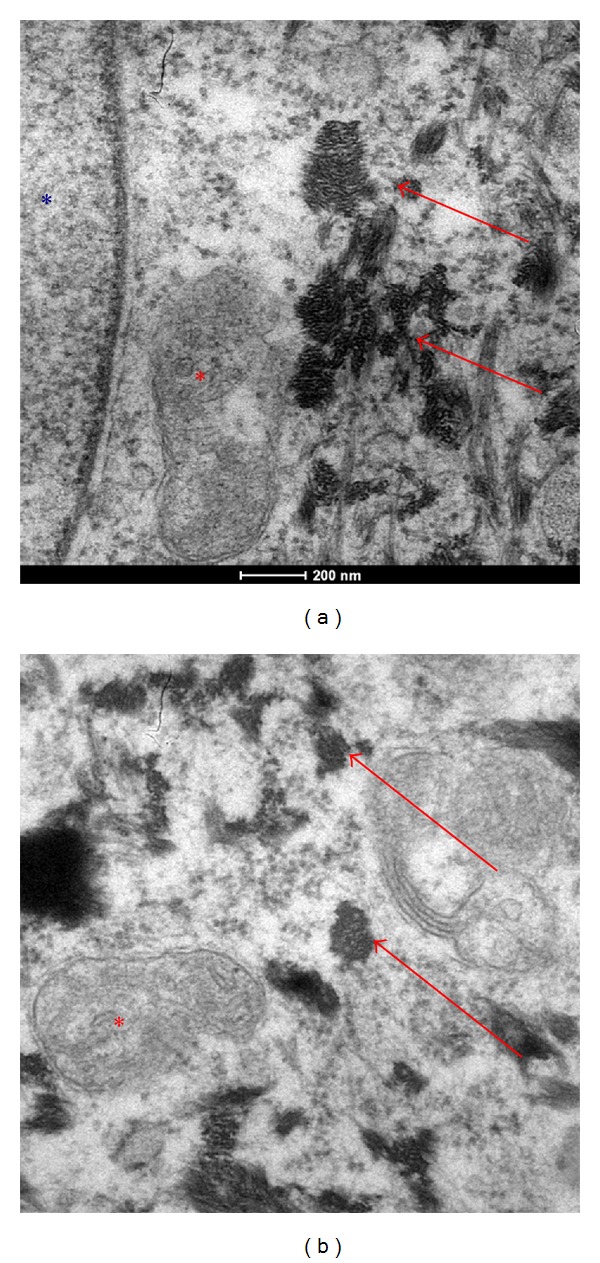
TEM images of AgNps (red arrows) in cytoplasmatic space of fibroblast present in a healed skin sample treated with AgNps based Medical devices. AgNP are closed to the mitochondria (red*); and not inside the mitochondria and inside the nuclei (indicated by blue*).

**Figure 7:**
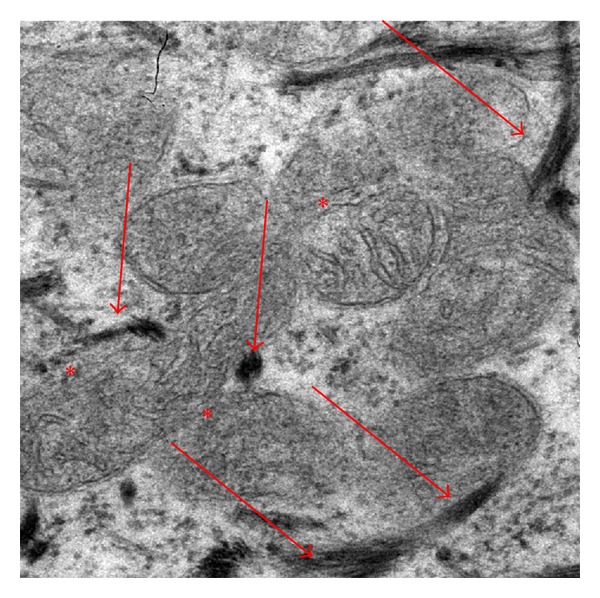
TEM images of AgNps (red arrows) in cyplosmasmatic space of fibroblast present in a healed skin sample treated with AgNP based Medical devices. AgNP (red arrows) are closed to the mitochondria (red*).

**Figure 8:**
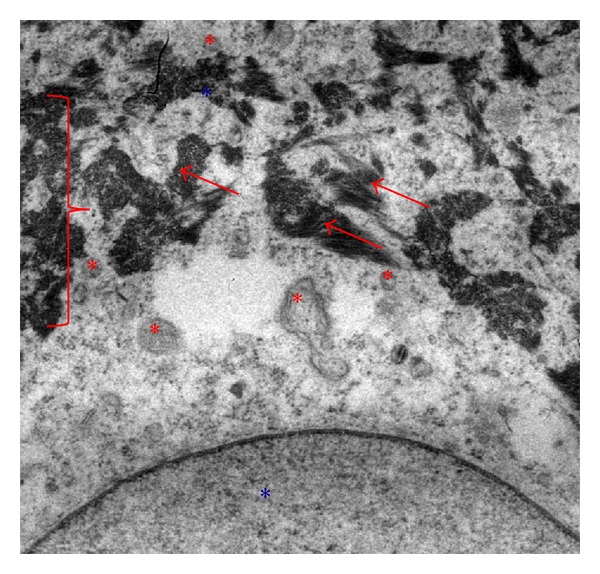
TEM images of AgNps (red arrows) in cytoplasmatic space of fibroblast present in a healed skin sample treated with AgNP based Medical devices. AgNps are closed to the outer membrane of the mitochondria that are organized in the perinuclear zone of the cytoplasm (square barked red), recruiting the larger quantity of AgNps present in the cytoplasm.

As regards intracellular localization of AgNps, their ability to form aggregates of about 200 nm (Figure 4, red arrows) was confirmed. They are absent from the cell nucleus, endoplasmic reticulum, or Golgi complex (Figure 5). They also formed agglomerates in the perinuclear region (Figure 5, red arrows). The transmission electron microscopy (TEM) analysis indicated the absence of AgNps (Figures 5 and 6 red arrows) inside the mitochondria (Figures 5 and 6 red*) and nucleus (Figure 5 blue*). AgNps are closed to the outer membrane of the mitochondria as it is well shown in Figure 7 red arrows (mitochondria red*). Mitochondria are organized in the perinuclear zone of the cytoplasm (Figure 8, square barked red), recruiting the larger quantity of AgNps (Figure 8 red arrows) present in the cytoplasm.

In these conditions, mitochondria are small (Figure 8 red*), round and in high number.

**Figure 9:**
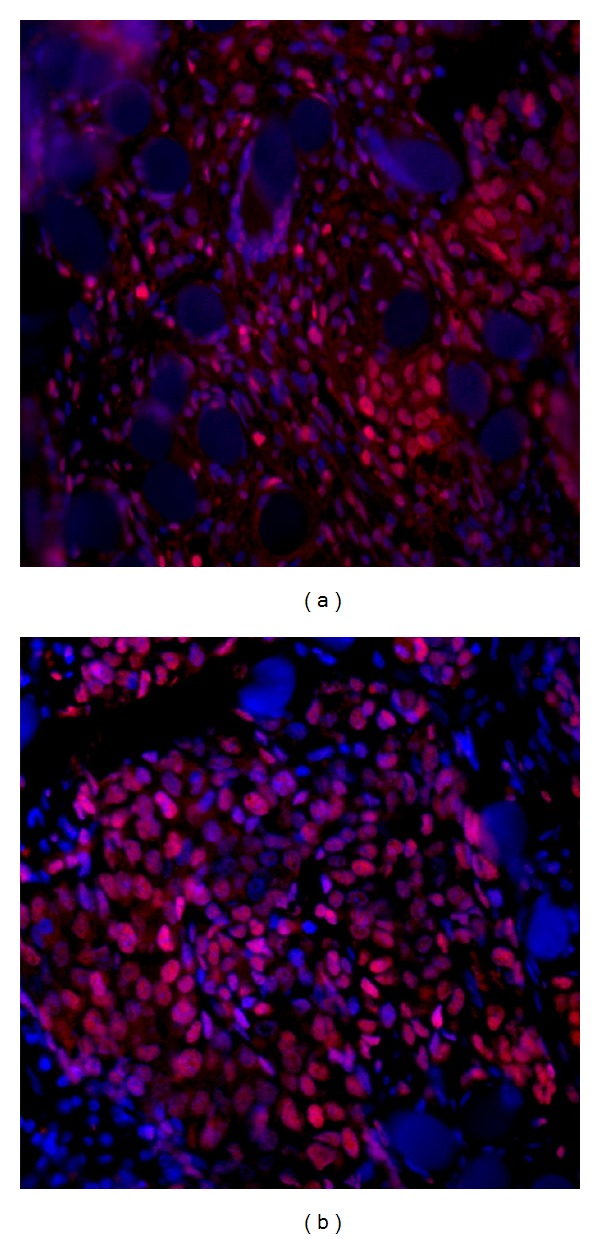
Dermal-like tissue reconstructed *in vitro*. Cells, visible thanks to the red staining of the nuclei, can be seen inside the collagen-based scaffold and appear to be organized in layers. (a) Un-treated control after 9 days from the beginning of the experiments; (b) treated control at nine days.

To investigate if Ag NPs could negatively affect cell survival, we evaluated their toxicity on fibroblasts *in vitro*. A collagen-based scaffold was employed as a support for a 3D cell culture of fibroblasts to obtain a dermal-like tissue. Morphological analyses were carried out to investigate nuclei morphology and cellular distribution within the scaffold. As reported in Figure 9(a) the dermal-like tissue appears as a multilayer of cells, where the fibroblasts are able to proliferate and fill the scaffolds during the course of the experiments. No signs of apoptosis were detected. The similar distribution of cells was seen in the Ag NP-treated samples (Figure 9(b)). Interestingly, despite the reduced mitochondrial functionality observed, the nuclei are still present and appear to be undamaged. There was no observable presence of apoptotic bodies or nuclear fragmentation.

**Figure 10:**
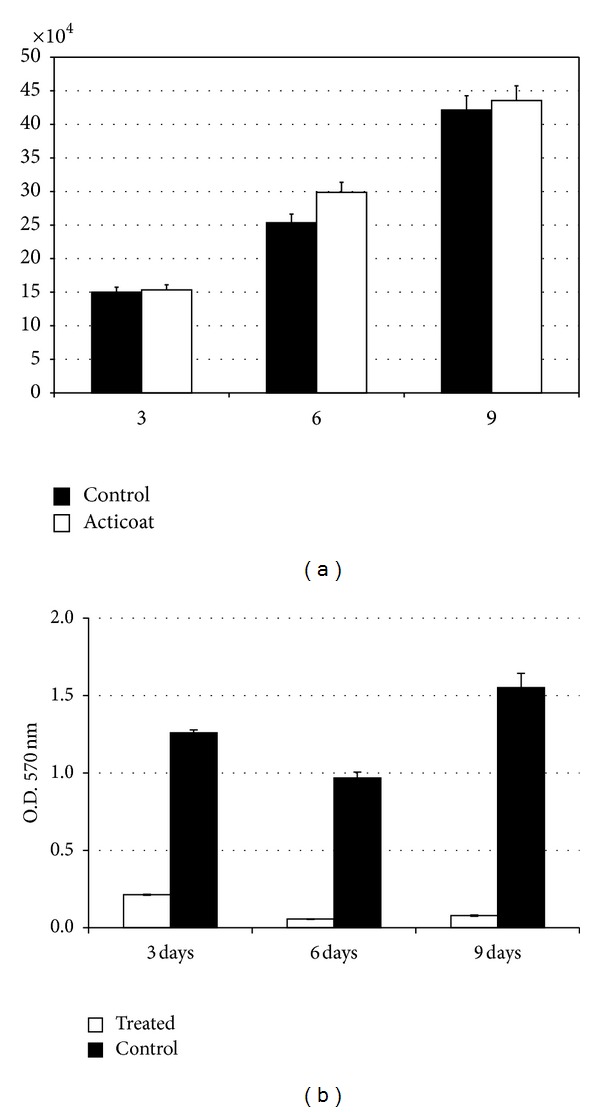
(a) Progression of cell growth in time in a 3D dermal-like tissue after Ag NP streatment (white) and in the control sample (black), mean value ± SD samples versus time. The count of the live cells in the sample is obtained as the sum of the live cells at various depths at each position. (b) MTT test for mitochondrial activity in a 3D dermal-like tissue after Ag NPs treatment (white) and in the control sample (black) mean value ± SD samples versus time. As it is well evident, when silver nanoparticles are present, no mitochondrial activity is detectable.

A quantitative comparison of the number of live cells in the treated and untreated 3D cell cultures is reported in Figure 10(a). The results show that the number of live cells increased with time at the same rate in both samples. The YO-PRO-1 assay showed that there were no apoptotic cells visible in the sample treated with Ag NPs.

At three, six, and nine days, MTT assays were carried out to assess the mitochondrial function in cells treated with Ag NPs. Cytotoxicity *in vitro *is usually estimated with the use of colorimetric tests; their principle is the reduction of tetrazolium salts, MTT (3-(4,5-dimethylthiazol-2-yl)-2,5-diphenyltetrazolium bromide) to formazan. The reduction is carried out by a mitochondrial reductase and is an indirect measure of cell population viability.

As reported in Figure 10(b), a time-dependent decrease in metabolic activity was observed in the cells treated with the Ag NP-based dressing. This confirms the ability of AgNPs to impair mitochondrial function. Examples of MTT test application for AgNps cytotoxicity (ROS) are present in every cell, being produced by the mitochondrial and cytoplasmic oxidation processes. Under environmental stress, the cell reacts by increased ROS generation, and this leads to imbalance between ROS generation and their neutralization by antioxidative enzymes and low molecular weight antioxidants, among others by glutathione. This disturbance of the redox equilibrium is defined as oxidative stress. Under conditions of oxidative stress the cell accumulates ROS, and the antioxidative response that follows involves modifications in signaling pathways. 

**Figure 11:**
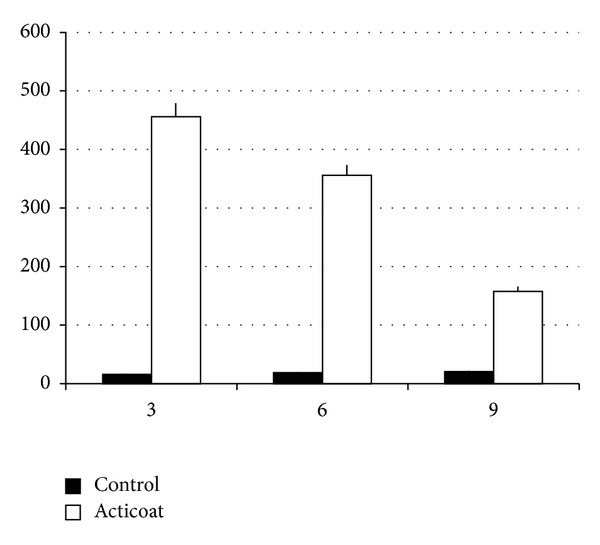
The ROS measurement in dermal-like tissue reconstructed *in vitro* with (white) and without NgNPs (black) treatment. As it is well evident, when silver nanoparticles are present, ROS activity is detectable.

In order to test if this damage could be correlated to a ROS production we tested ROS generations. As reported in Figure 11 there is a ROS production in presence of AgNPs. This production decreases in time, according to the ability of the mitochondria to capture AgNPs.

## 4. Conclusions

ROS increase due to nanoparticle treatment has been shown to be the key factor in the biological effects *in vivo* and *in vitro* [23–25]. From our TEM microphotographs it can be judged that AgNps accumulate outside the mitochondria. It is possible that this is the direct cause of mitochondrial damage and the disturbed function of the respiratory chain resulting in ROS generation and oxidative stress. No AgNps were detected inside the nucleus, and no fragmented nuclei were observed. Despite the presence of AgNps inside the cytoplasm, the nuclear membrane is intact and is round in shape. The nucleolus visible confirms that the chromatin is not condensed, but that it has a structure that allows transcription to proceed. We hypothesize that once the AgNps have been released into the cytoplasm, they generate ROS.

We speculate, then, that mitochondria are moved around the nucleus to act as a physical-chemical barrier to prevent ROS and AgNps from reaching the nuclear membrane. If the mitochondrial membrane breaks down due to the action of ROS, antioxidative enzymes, such as mitochondrial superoxide dismutase (mtSOD), catalase, glutathione peroxidase, and thioredoxin peroxidase [26, 27], are released from the mitochondria into the cytoplasm to quench the ROS.
